# Why Does Cross-Sectional Analyst Coverage Incorporate Market-Wide Information?

**DOI:** 10.3390/e26040285

**Published:** 2024-03-26

**Authors:** Yunfei Hou, Changsheng Hu

**Affiliations:** School of Economics and Management, Wuhan University, Wuhan 430072, China; 00001860@whu.edu.cn

**Keywords:** analyst coverage, exponential distribution, market-wide information, maximum entropy

## Abstract

This paper shows that the empirical distribution of cross-sectional analyst coverage in China’s stock markets follows an exponential law in a given month from 2011 to 2020. The findings hold in both the emerging (Shanghai) and the developed market (Hong Kong). Moreover, the unique distribution parameter (i.e., mean) is directly related to the amount of market-wide information. Average analyst coverage exhibits a significant negative predictive power for stock-market uncertainty, highlighting the role of security analysts in diminishing the total uncertainty. The exponential law can be derived from the maximum entropy principle (MEP). When analysts, who are constrained by average ability in generating information (i.e., the first-order moment), strive to maximize the amount of market-wide information, this objective yields the exponential distribution. Contrary to the conventional wisdom that security analysts specialize in the generation of firm-specific information, empirical findings suggest that analysts primarily produce market-wide information for 25 countries. Nevertheless, it remains unclear why cross-sectional analyst coverage reflects market-wide information, this paper provides an entropy-based explanation.

## 1. Introduction

Sell-side analysts are among the most influential information producers in financial markets, playing a crucial role for both investors and managers. Numerous empirical studies have documented the impact of analyst attention (i.e., coverage) on investors’ decisions (e.g., [1,2,3]), as well as on corporate activities such as investment and financing, innovation (e.g., R&D), acquisition, and earnings management (e.g., [4,5,6,7]).

However, contrary to the conventional wisdom that security analysts specialize in collecting and disseminating firm-specific information, empirical findings indicate that analysts predominantly generate market-wide information for 25 countries [8,9]. It remains unclear why analyst reports reflect market-wide information rather than firm-specific information. To deeply understand the underlying mechanism, we examine the empirical distribution of cross-sectional analyst coverage.

This paper illustrates that the empirical distribution of analyst coverage for China’s listed companies maintains an exponential structure in a given month from 2011 to 2020. Our findings are consistent in both the Shanghai and Hong Kong stock markets. Since the system information of exponential distribution can be fully characterized by the mean value, we expect that aggregate analyst coverage can help reduce total uncertainty for both investors and managers. This paper provides evidence that aggregate (or average) analyst coverage, denoted by $\lambda^{-1}$, exhibits a strong negative predictive power for stock-market uncertainty.

Given that cross-sectional analyst coverage is exponentially distributed, a natural question is how the exponential structure occurs. Motivated by [10], who draw on the maximum entropy principle (MEP) to interpret the asymmetric Laplace-shaped distribution of Tobin’ *Q*, we utilize the MEP to derive the exponential distribution of analyst coverage. The central idea is that when analysts, who are constrained by limited average ability in producing information (i.e., the first-order moment), aim to maximize the amount of market-wide information, this objective can yield an exponential distribution. To the best of our knowledge, it has not been documented in the previous literature that cross-sectional analyst coverage follows an exponential law. Our study is the first to provide an explanation for why analyst coverage predominantly incorporates market-wide information.

Our findings have some important implications. Firstly, sell-side analysts primarily generate market-wide information. Secondly, almost all relevant studies claim that coverage proxies should be used in logarithmic form to mitigate the influence of outliers; however, our results suggest that the exponential structure would be distorted when logarithmic transformation is performed. Thirdly, because more than half of the firms have similar coverage, future research should be especially cautious when using coverage proxies in cross-sectional and particularly in portfolio analyses.

The rest of this paper is organized as follows. Section 2 reports the empirical distribution of cross-sectional analyst coverage in Chinese stock markets. Section 3 provides a potential mechanism for the exponential law. In Section 4, we make concluding discussions.

## 2. Exponentially Distributed Cross-Sectional Analyst Coverage

Assuming a random variable *X* is exponentially distributed, it can be written as,

(1) $$\mathrm{Exp}\;\left[\lambda \right]:\;f\left[x\right]=\lambda e^{-\lambda x}$$

where x>0,λ>0, f[x] is the probability density function (PDF) with $E\left[X\right]=\sqrt{D[X]}=\lambda^{-1}$. Empirically, it is better to work with the complementary cumulative distribution function, i.e., CCDF: $P\left[X>x\right]=e^{-\lambda x}$. The parameter λ can be obtained by the slope of the straight line on a semi-log scale: log(CCDF) vs. *x*.

We examine the empirical distribution of cross-sectional analyst coverage in China’s stock markets. The sample consists of all common stocks listed on the Shanghai Stock Exchange (SSE), the Shenzhen S tock Exchange (SZSE), and the Hong Kong Stock Exchange (HK) from January 2011 to January 2020. The data are all from the Wind database. Table 1 presents the descriptive statistics of the monthly coverage data (i.e., *x*) for the three stock markets.

### 2.1. Evidence from the Shanghai, Shenzhen, and Hong Kong Stock Markets

**Figure 1:**
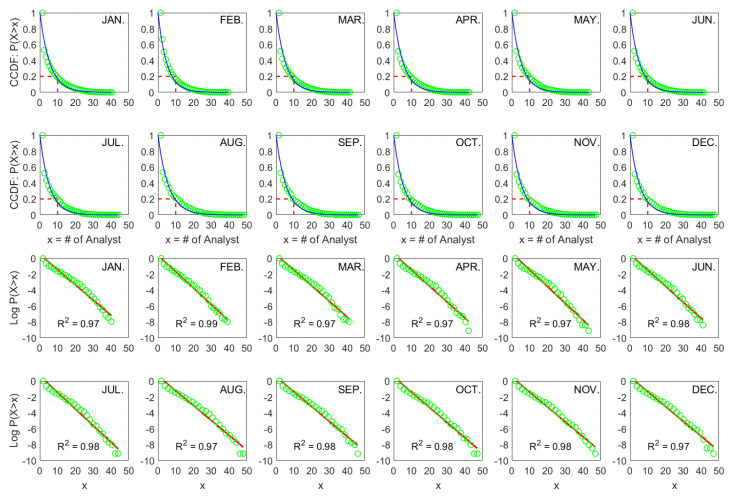
Exponential fits of complementary cumulative distribution functions (CCDFs) of cross-sectional analyst coverage (*x*) on the linear (blue) and semi-log (red) scales for each month from 2011 to 2020 in the Shanghai stock market (SSE).

The monthly results are shown in Figure 1. Two things are evident. First, the CCDFs show the highly skewed structure of cross-sectional analyst coverage. We observe that the percentage of firms with coverage below 10 is about 80%, i.e., a very high proportion of firms are covered by a small number of analysts, while certain firms have substantial coverage. Second, and most importantly, the empirical distributions are well fitted by Exp [λ] on both linear (CCDF vs. x) and semi-log (log CCDF vs. x) scales, where exponential fits are denoted as blue and red lines, respectively.

One might be intrigued by the goodness of fit; we thus present the regression $R^{2}$ in each semi-log subplot. On average, the $R^{2}$ is as high as 98% for the period 2011–2020. Notably, we do not exclude any coverage data based on any firm characteristics, which ensures that our findings provide a clean and complete picture of attention allocation structure of sell-side analysts.

**Figure 2:**
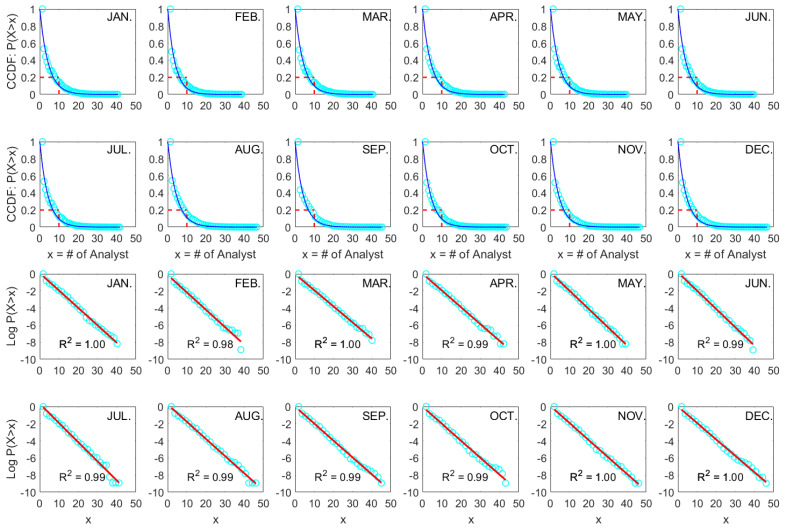
Exponential fits of complementary cumulative distribution functions (CCDFs) of cross-sectional analyst coverage (*x*) on the linear (blue) and semi-log (red) scales for each month from 2011 to 2020 in the Hong Kong stock market (HK).

Furthermore, as shown in Figure 2, a consistent exponential pattern of cross-sectional analyst coverage is evident for the Hong Kong stock market (HK), indicating that our findings are consistent in both emerging and developed stock markets.

This paper argues that using coverage proxy to measure the amount of firm-specific information poses a serious inference problem. It is impossible that the amounts of information for firms with zero-coverage are exactly equal. After excluding firms with no coverage, 80% of firms have almost the same small amount of analyst coverage. In any case, one cannot state that the majority of firms with approximate levels of coverage have similar firm-specific information environments.

**Figure 3:**
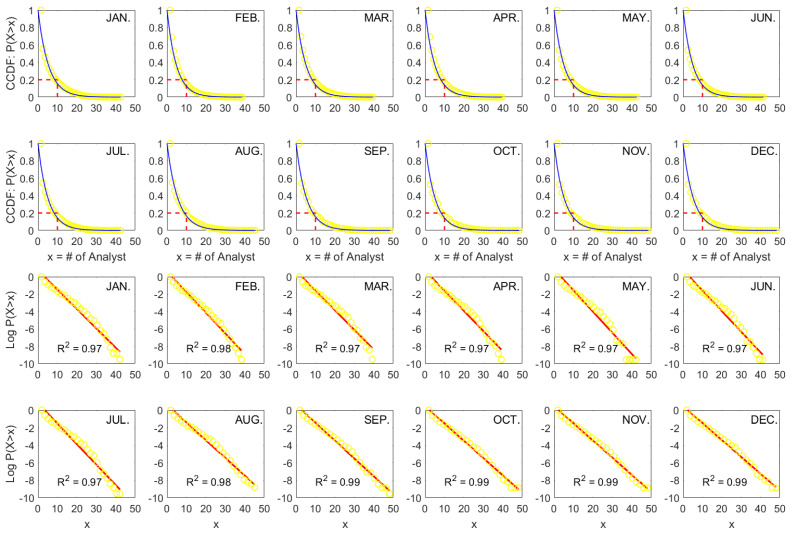
Exponential fits of complementary cumulative distribution functions (CCDFs) of cross-sectional analyst coverage (*x*) on the linear (blue) and semi-log (red) scales for each month from 2011 to 2020 in the Shenzhen stock market (SZSE).

**Figure 4:**
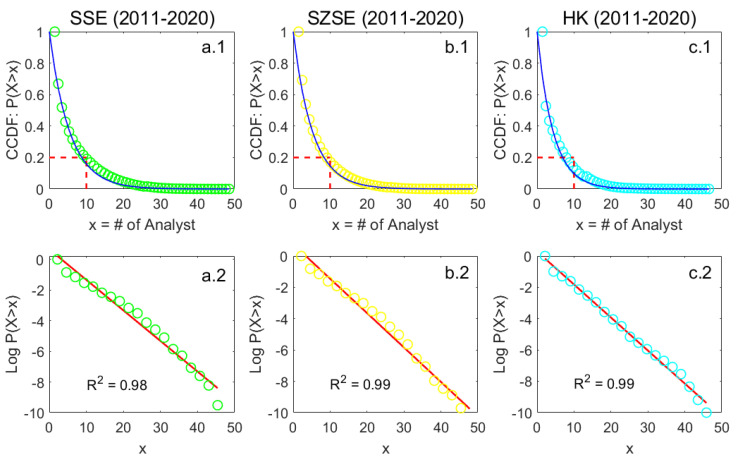
Exponential fits of complementary cumulative distribution functions (CCDFs) of cross-sectional analyst coverage (*x*) on the linear (blue) and log-linear (red) scales during the period 2011–2020 in the SSE (**a.1**,**a.2**), SZSE (**b.1**,**b.2**), and HK (**c.1**,**c.2**) stock markets.

**Figure A1:**
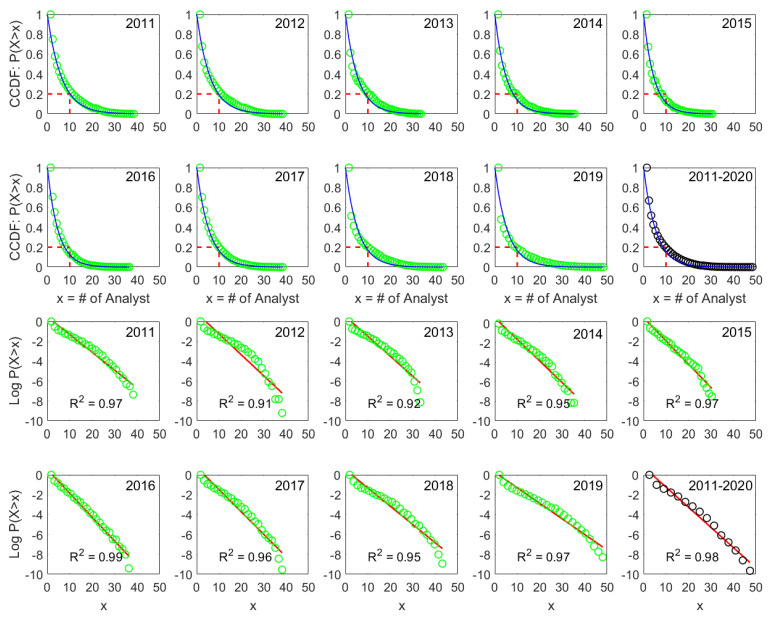
Exponential fits of complementary cumulative distribution functions (CCDFs) of cross-sectional analyst coverage (*x*) on the linear (blue) and semi-log (red) scales for each year from 2011 to 2020 in the Shanghai stock market (SSE).

By comparing the fitting results of the Shanghai (SSE) and the Hong Kong (HK) stock markets, it is observed that the HK data conform well to the exponential law, while the SSE data deviate from a linear relationship when expressed in log-linear form, especial as shown in Figure A1. It is an important question whether an exponential is the proper fit for the emerging markets such as the SSE. To address this concern, we further supplement the investigation with additional data from the Shenzhen stock market (SZSE). The monthly results for the SZSE are presented in Figure 3, showing a similar exponential pattern to that observed in the SSE. Figure 4 demonstrates that for large samples, exponential fitting performs better on both linear and log-linear scales in the SSE (a), SZSE (b), and HK (c) stock markets. The empirical distributions of coverage data deviate slightly from the exponential law in the long run. In other words, the deviation can, to some extent, be treated as a short-term phenomenon.

The goodness of fit is high for the period 2011–2020, reaching 0.98, 0.99, and 0.99, respectively. However, a closer inspection in the performance of exponential fitting reveals that the results for the SSE and SZSE stock markets do not follow a simple exponential law. There is a change in the gradient midway along the abscissa visible in all the graphs. Hence, we will conduct a detailed and in-depth evaluation of curve fitting in the next section.

### 2.2. Difference in Exponential Fitting between SSE, SZSE, and HK

**Figure 5:**
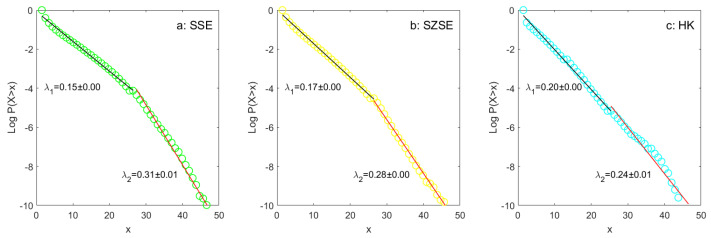
Exponential fits of CCDFs in two scaling regimes (denoted as $\lambda_{1}$ and $\lambda_{2}$) for cross-sectional analyst coverage (*x*) of the SSE (**a**), SZSE (**b**), and HK (**c**) stock markets during the period 2011–2020.

In order to clearly observe the change in the gradient at the middle position of the abscissa, we divided the bins more densely when calculating CCDFs, increasing the number of bins from 20 to 50. Figure 5 assesses the exponential fits for cross-sectional analyst coverage (*x*) of the SSE (a), SZSE (b) and HK (c) stock markets in two scaling regimes, denoted as $\lambda_{1}$ and $\lambda_{2}$. For the SSE and SZSE, even over the long term, the deviations in the fitted values of the two regimes are significant. Subplot c shows that the deviation is relatively small in the HK stock market.

Furthermore, we conduct statistical tests on this difference between SSE, SZSE, and HK. Panel A and B of Table 2 show the comparison results. The monthly fitted parameters, namely λ, $\lambda_{1}$, $\lambda_{2}$, and $\bar{\lambda }$, are displayed in the first four columns. The last four columns illustrate the deviations between these scaling regimes. On average, we conclude that there are statistically significant breakpoints in the exponential fitting for the SSE and SZSE, while the cross-sectional analyst coverage for the HK market exhibits a simple scaling similar to an exponential law.

The higher the costs of gathering information, the greater the potential resource constraints faced by security analysts. The relatively larger $\lambda_{1}$ for firms with less coverage may imply that analysts need to spend more time and effort in studying and covering these companies, which are smaller in size and more prone to earning manipulation (e.g., [4,7]). Intuitively, this could be attributed to the lower market visibility of these companies or the relatively limited availability of relevant market-wide information about them. Deviation of exponential fitting across distinct scaling regimes is observed in the SSE and SZSE stock markets but not in the HK market, indicating a significant difference between SSE, SZSE, and HK. More importantly, can the difference in curve-fit bias serve as one of the distinguishing factors between emerging and developed stock markets? To effectively address this question, future research should provide richer comparative evidence across countries.

### 2.3. Predicting Stock-Market Uncertainty

#### 2.3.1. Distribution Changes during the Period 2011–2020

**Figure 6:**
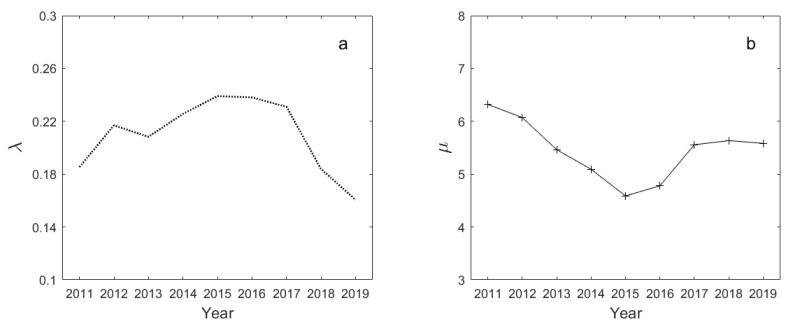
Fitted values of parameter λ (**a**) and sample mean μ (**b**) for cross-sectional analyst coverage of the Shanghai stock market (SSE) for each year from 2011 to 2020.

Before demonstrating the negative predictive power of time-varying market information flows measured by $\lambda^{-1}$ on total uncertainty, it is necessary to illustrate how the statistical properties of the distribution evolved during the period. Figure 6 shows the fitted values of parameter λ (a) and sample mean μ (b) for cross-sectional analyst coverage of the Shanghai stock market (SSE) from 2011 to 2020. Additionally, as illustrated in Figure A1 in the Appendix A, it is observed that the empirical distribution maintains a stable exponential pattern for each year.

An interesting fact is that the total amount of market information provided by sell-side analysts decreased significantly in 2015, as seen in Figure 6, and the maximum coverage number was the lowest during the sample period (see Figure A1). As we all know, the Chinese stock market experienced two crash crises in 2015 and early 2016.

Given the finding that sell-side analysts mainly generate market-wide information (see [8,9]), we go one step further and directly examine whether aggregate analyst coverage is related to stock-market uncertainty. In this paper, aggregate analyst coverage is denoted by the inverse of parameter λ, since $\lambda^{-1}$ fully characterizes the mean value of cross-sectional analyst coverage. The finding has many implications, and the most critical one is that aggregate analyst coverage exhibits a negative predictive power for stock-market uncertainty. If analysts have no advantage over insiders in generating firm-specific information, focusing on providing market-wide information becomes an inevitable choice. Next, we test the hypothesis that aggregate analyst coverage (denoted by $\lambda^{-1}$) can help reduce the expected total uncertainty.

#### 2.3.2. Predictive Regression Results

We examine the forecasting power of aggregate analyst coverage denoted by $\lambda^{-1}$ for stock-market uncertainty based on the following time-series predictive regression,

(2) $$U_{t,t+h}^{j}=\alpha +\delta \lambda_{t}^{-1}+\mathrm{Controls}+\varepsilon_{t,t+h}$$

where $U_{t,t+h}^{j}$ is the stock-market uncertainty over the prediction horizon *h*, where h=1,6 and 12 months, and *j* denotes two uncertainty proxies, which are market-level cash-flow volatility (i.e., CFV) and investor search volume (i.e., Search). $\lambda_{t}^{-1}$ is the inverse of the parameter of exponentially distributed analyst coverage in month *t*, which captures continuous-varying marker information flows. We also control for a linear time trend and lagged stock-market uncertainty up to five lags.

Our analysis in Table 3 is motivated by [12], who theoretically show that the expected amount of information generated equals the expected reduction in uncertainty. The idea is consistent with the foundational work in information theory [13]. Concretely, we construct two proxies for stock-market uncertainty that are closely related to managers and investors, respectively. One is market-level cash flow volatility, i.e., ${\mathrm{CFV}}_{t}$ (e.g., [14,15]), which directly measures the uncertainty of operating management. The other is investor search volume denoted as ${\mathrm{Search}}_{t}$. This is because theories of rational information acquisition predict that investors’ information-search demand increases in the uncertainty about asset payoffs (e.g., [16,17,18]).

Table 3 suggests that an increase in aggregate analyst coverage is associated with an expected decrease in manager uncertainty (Panel A) and investor uncertainty (B), respectively. The results remain robust after controlling for a linear time trend and lagged stock-market uncertainty up to five lags in models (2), (4), and (6). In summary, we conclude that aggregate analyst coverage can alleviate total uncertainty for both investors and managers. Moreover, a higher aggregate analyst coverage also precedes lower market-wide cash holdings and the absolute magnitude of unexpected earnings, as well as greater capital expenditures and long-term debt. For more comprehensive and detailed evidence, see [20,21].

## 3. MEP Generates the Exponential Distribution

Finally, this paper attempts to provide a potential generation mechanism for the exponentially distributed analyst coverage. Motivated by [10], who draw on the maximum entropy principle (MEP) to interpret the asymmetric Laplace distribution of Tobin’ *Q*. In what follows we utilize the MEP to derive the exponential law. The idea of MEP is that in making inferences on the basis of partial information, we must use the probability distribution that achieves maximum entropy [22].

The concept of maximum-entropy optimization has been advocated in economic analysis (see [23,24]). In an economic context, examples of constraints that we impose include budget constraints, non-negativity of prices, average corporate profit rate (e.g., [10]), and behavioral constraints (e.g., limited attention in this paper).

Suppose that our only knowledge of analysts coverage distribution is the mean value (i.e., limited average-ability in producing information). Mathematically, the maximum entropy problem subject to the first-order moment constraint is as follows,

(3) $$\begin{array}{c} \max_{\left\{f\left[x\right]\geq 0|x\in R^{+}\right\}}\;h\left(f\left[x\right]\right)=-\int_{x}f\left[x\right]\log f\left[x\right]dx\;\;s.t.\;\;\int_{x}f\left[x\right]dx=1,\;\;E\left[X\right]=\int_{x}xf\left[x\right]dx=\mu_{x} \end{array}$$

The Lagrangian associated with this programming problem is,

(4) $$J\left(f\left[x\right]\right)=-\int_{x}f\left[x\right]\log f\left[x\right]dx+\lambda_{1}\left(\int_{x}f\left[x\right]dx-1\right)+\lambda_{2}\left(\int_{x}xf\left[x\right]dx-\mu_{x}\right)$$

Taking the first-order condition and solving for f[x] yields,

(5) $$f^{*}\left[x;\lambda \right]=\lambda e^{-\lambda x},\;\;E\left[X\right]=\lambda^{-1}$$

The solution is an exponential distribution, which is fully described by the unique parameter λ. The first-order moment constraint condition is natural due to the fact that analysts have limited average capability in providing information. This entropy maximization property is perhaps the main reason why we encounter exponential distributions so frequently in mathematics and physics. One might ask what entropy, i.e., h(f[x]), means in our case. In the Shannon theory, this answer is clear. The average amount of information is given by the information entropy, i.e., h(f[x]), as Shannon pointed out [13]. Because analyst coverage can be viewed as a standard information source, maximizing the objective function is equivalent to maximizing the amount of market-wide information. Similarly, supposing we do not have any knowledge about distribution, the solution of maximum-entropy optimization is the Uniform distribution. In this case, when each firm has the same coverage (no firm-specific information), the amount of market-wide information attains the maximum value. In both cases, sell-side analysts generate market-wide information, not firm-specific information.

## 4. Conclusions and Discussion

Contrary to the conventional wisdom that sell-side analysts specialize in collecting and disseminating firm-specific information, it is found that analysts predominantly generate market-wide information for 25 countries (see [8,9]). However, it is still unclear why analyst coverage reflects market-wide information rather than firm-specific information. To understand the underling reasons, we examine the empirical distribution of cross-sectional analyst coverage.

This paper shows the following key findings: (i) the empirical frequency distribution of cross-sectional analyst coverage follows an exponential law, observed in both the Shanghai and Hong Kong stock markets; (ii) an increase in aggregate analyst coverage is associated with an expected decrease in total uncertainty; (iii) when analysts, who are constrained by limited average ability in producing information (i.e., the first-order moment), strive to maximize the amount of market-wide information, this objective yields the exponential distribution.

In summary, our findings offer two main insights. First, sell-side analysts in China’s stock markets predominantly contribute to market-wide information, illuminating their role in mitigating total uncertainty. Second, future research should be extra cautious when using coverage proxies in cross-sectional and portfolio analyses due to potential inference issues. Strictly speaking, it is advisable to carefully re-evaluate previous cross-sectional findings, if feasible.

## Figures and Tables

**Table 1:** Descriptive statistics of monthly coverage data.

	Mean	S.D.	Min	Max	Med.	Skew.	Kurt.	No.
$x_{\mathrm{SSE}}$	5.44	6.24	1	49	3	1.95	6.93	108,282
$x_{\mathrm{SZSE}}$	5.19	5.64	1	49	3	1.98	7.34	165,678
$x_{\mathrm{HK}}$	4.30	4.99	1	47	2	2.09	8.62	88,794

**Table 2:** The difference in monthly fitted parameters between SSE, SZSE and HK.

		λ	$\lambda_{1}$	$\lambda_{2}$	$\bar{\lambda }$		($\lambda -\lambda_{1}$)	($\lambda -\lambda_{2}$)	($\lambda_{1}-\lambda_{2}$)	($\lambda -\bar{\lambda }$)
Panel A. Monthly exponential fitting for SSE and SZSE										
$x_{\mathrm{SSE}}$		0.1876	0.1484	0.2530	0.2007		0.04 ***	−0.07 ***	−0.10 ***	−0.01 ***
NW *t*							[8.75]	[−6.56]	[−7.54]	[−3.84]
$x_{\mathrm{SZSE}}$		0.2061	0.1680	0.2712	0.2196		0.04 ***	−0.07 ***	−0.10 ***	−0.01 ***
NW *t*							[11.6]	[−7.55]	[−8.83]	[−4.46]
Panel B. Monthly exponential fitting for HK										
$x_{\mathrm{HK}}$		0.2549	0.2041	0.3149	0.2595		0.05	−0.06	−0.11	−0.00
NW *t*							[1.58]	[−1.33]	[−1.44]	[−0.69]

Note: $\bar{\lambda }=\left(\lambda_{1}+\lambda_{2}\right)/2$. The sample period is from 2011 to 2020. The Newey–West (NW) *t*-statistics in brackets are adjusted for autocorrelation and heteroskedasticity [11]. *** indicates statistical significance at the 1% level.

**Table 3:** Diminishing the expected stock-market uncertainty.

Panel A. Predicting future manager uncertainty proxied by cash-flow volatility ${\mathrm{CFV}}_{t,t+h}$						
	(1)	(2)	(3)	(4)	(5)	(6)
	${\mathrm{CFV}}_{t,t+1}$	${\mathrm{CFV}}_{t,t+1}$	${\mathrm{CFV}}_{t,t+6}$	${\mathrm{CFV}}_{t,t+6}$	${\mathrm{CFV}}_{t,t+12}$	${\mathrm{CFV}}_{t,t+12}$
$\lambda_{t}^{-1}$	−1.38 ***	−0.16 **	−1.42 ***	−0.52 ***	−1.42 ***	−0.61 ***
	[−8.29]	[−2.27]	[−8.68]	[−3.57]	[−9.47]	[−5.25]
Trend	No	Yes	No	Yes	No	Yes
Lagged CFV	No	Yes	No	Yes	No	Yes
N	108	108	102	102	96	96
${\mathrm{adj}.R}^{2}$	0.31	0.86	0.42	0.82	0.50	0.86
ADF.prob	1 × $10^{-3}$	1 × $10^{-3}$	5 × $10^{-3}$	1 × $10^{-3}$	2 × $10^{-3}$	5 × $10^{-3}$
Panel B. Predicting future investor uncertainty proxied by information demand ${\mathrm{Search}}_{t,t+h}$						
	${\mathrm{Search}}_{t,t+1}$	${\mathrm{Search}}_{t,t+1}$	${\mathrm{Search}}_{t,t+6}$	${\mathrm{Search}}_{t,t+6}$	${\mathrm{Search}}_{t,t+12}$	${\mathrm{Search}}_{t,t+12}$
$\lambda_{t}^{-1}$	−0.40 ***	−0.34 ***	−0.39 ***	−0.20 ***	−0.34 ***	−0.26 ***
	[−4.65]	[−4.04]	[−9.22]	[−7.16]	[−8.87]	[−6.22]
Trend	No	Yes	No	Yes	No	Yes
Lagged Search	No	Yes	No	Yes	No	Yes
N	108	108	102	102	96	96
${\mathrm{adj}.R}^{2}$	0.22	0.58	0.40	0.65	0.42	0.71
ADF.prob	1 × $10^{-3}$	1 × $10^{-3}$	1 × $10^{-2}$	5 × $10^{-3}$	1 × $10^{-2}$	1 × $10^{-2}$

Note: This table reports time-series predictive regressions of expected stock-market uncertainty on aggregate analyst coverage denoted as $\lambda_{t}^{-1}$. The dependent variables are the future market-level cash-flow volatility, i.e., ${\mathrm{CFV}}_{t,t+h}$ (Panel A) and investor search volume, i.e., ${\mathrm{Search}}_{t,t+h}$ (Panel B), which measure the manager and investor uncertainty, respectively. Controls in models (2), (4), and (6) include a linear time trend and lagged market-uncertainty up to five lags. To address concerns about spurious regression, we utilize the ADF tests. The sample period is 2011−2020. The *t*-statistics in brackets are adjusted for heteroskedasticity [19]. ** and *** indicate significance at the 5% and 1% level, respectively.

## Data Availability

All data are from the Wind database.
